# Novel Associations Within the Tumor Microenvironment: Fibulins Meet ADAMTSs

**DOI:** 10.3389/fonc.2019.00796

**Published:** 2019-08-22

**Authors:** Tania Fontanil, Yamina Mohamedi, Teresa Cobo, Santiago Cal, Álvaro J. Obaya

**Affiliations:** ^1^Departamento de Bioquímica y Biología Molecular, Universidad de Oviedo, Oviedo, Spain; ^2^Departamento de Investigación, Instituto Órdoñez, Oviedo, Spain; ^3^Instituto Universitario de Oncología, IUOPA, Universidad de Oviedo, Oviedo, Spain; ^4^Departamento de Cirugía y Especialidades Médico-Quirúrgicas, Instituto Asturiano de Odontología, Universidad de Oviedo, Oviedo, Spain; ^5^Departamento de Biología Funcional, Área de Fisiología, Universidad de Oviedo, Oviedo, Spain

**Keywords:** fibulin, ADAMTS, cancer, microenvironment, extracelular matrix

## Abstract

The maintenance of tissue homeostasis in any organism is a very complex and delicate process in which numerous factors intervene. Cellular homeostasis not only depends on intrinsic factors but also relies on external factors that compose the microenvironment or cellular niche. Thus, extracellular matrix (ECM) components play a very important role in maintaining cell survival and behavior, and alterations in the ECM composition can lead to different pathologies. Fibulins and ADAMTS metalloproteases play crucial roles in the upkeep and function of the ECM in different tissues. In fact, members of both of these families of secreted multidomain proteins can interact with numerous other ECM components and thus shape or regulate the molecular environment. Individual members of both families have been implicated in tumor-related processes by exhibiting either pro- or antitumor properties. Recent studies have shown both an important relation among members of both families and their participation in several pathologies, including cardiogenesis or cancer. In this review, we summarize the associations among fibulins and ADAMTSs and the effects elicited by those interactions on cellular behavior.

## Cellular Microenvironment

The extracellular matrix (ECM) is a complex three-dimensional network of proteins and carbohydrates that serves as a support for cells and tissues (1). The cellular function of the ECM makes it a dynamic and versatile compartment through the modulation of the production, degradation, and remodeling of all its components, thus participating in organ development, function, and repairment (2).

The ECM is mainly composed of highly modified proteins, which contributes to its specialization in each organ. In general, the main components of the ECM are macromolecules and fibrous proteins as well as a wide variety of enzymes, including proteases, which are involved in processes such as the maturation, assembly, and renewal of ECM components. Thus, the ECM is responsible for maintaining the homeostasis of each individual tissue and, therefore, of the whole organism. In addition, interactions among ECM components help to mold the cellular microenvironment depending on the functional requirements of the organism (3). In particular, the participation of proteases in ECM processes implies irreversible modifications of their substrates, and proteases are involved in a multitude of crucial processes, such as bone remodeling (4), cardiogenesis (5), or even neural development (6). Notably, proteolytic activity can also be modified by a myriad of cofactors, inhibitors, and other modulatory factors. These elements, with the complete substrate repertoire of a particular protease, constitute proteolytic systems (7). Thus, importantly, substrate identification is one of the most important challenges in characterizing a proteolytic enzyme under both normal conditions and pathological alterations. In this context, instances of non-specificity, in which the same protease can process multiple substrates, or redundancy, in which some proteins are targets of more than one protease, can be found. Both situations show that proteolytic systems contain more information than the simple sum of their individual components; thus, each physiological or pathological context can be understood only as the result of the interaction of all ECM components.

In this review, we summarize the increasing number of investigations describing the associations between two families of ECM proteins: ADAMTS metalloproteases and fibulins. Different members of both families have been shown to interact and, through their interaction, modulate biological processes such as organogenesis and cancer development.

## Fibulins

**Figure 1:**
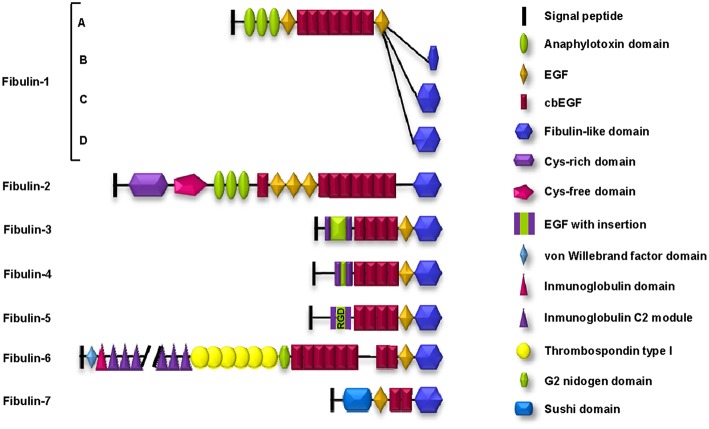
Schematic representation of the structure and motifs composition of the fibulin family of ECM proteins. Fibulin-5 contains an evolutionarily conserved integrin-binding sequence (RGD: arginine-glycine-aspartic acid) in the EGF module.

Fibulins are a main component of elastic fibers in the ECM that endow tissues with the ability to contract after stretching, a property called elastogenesis (8). Elastic fibers are basically composed of two different components: an amorphous elastin core, which is immersed within a fibrillar scaffold composed of microfibrils. In addition, the association of the elastin core with the microfibrils requires the participation of many proteins, among which are associated glycoproteins, latent TGF-β binding proteins, interfacial proteins, lysyl oxidases, and members of the fibulin family (9). The fibulin family comprises seven extracellular glycoproteins, all of which include a fibulin-type globular domain, which is also known as domain III, at the carboxy (C)-terminus (10). This common domain is preceded by domain II, which includes a variable number of epithelial growth factor (EGF)-like modules, some of which contain a calcium-binding consensus sequence; these domains are known as calcium-binding EGF (cbEGF)-like domains. Finally, domain I is located at the amino-terminus and shows the highest structural variability among the family members (11) (Figure 1).

By their architecture, mammalian fibulins can be classified into two subgroups (12). Subgroup I comprises fibulin-1 and fibulin-2 (100 and 200 kDa, respectively) and is characterized by the three anaphylatoxin modules in domain I. In addition, fibulin-2 differs from fibulin-1 because it contains two regions in domain I with additional differences: one rich in cysteines (the *cys-rich* domain) and the other lacking this amino acid (the *cys-free* domain).

Subgroup II is formed by the rest of the members but also shows important differences. Fibulin-3, fibulin-4, and fibulin-5 are also known as short fibulins since their molecular weights range from 50 to 60 kDa. Fibulin-6 is the largest fibulin in the family because it possesses an important number of different motifs in domain I: a von Willebrand factor motif, 44 immunoglobulin C2 modules, and 6 thrombospondin type I (TSP-1) motifs. Fibulin-7 is characterized by a “sushi” motif in domain I, which is involved in protein-protein interactions (13) (Figure 1).

## The ADAMTS Family

The ADAMTS family, whose acronym comes from “A Disintegrin And Metalloprotease with ThromboSpondin domains,” consists of ECM-secreted enzymes whose main characteristic is a set of different adhesion motifs together with a proteolytic motif within the same protein unit. ADAMTSs belong to the metzincin superfamily; thus, their protease domains share great similarity with those of the other members of this family. In this superfamily of proteases, the proteolytic domain is characterized by a zinc atom at the active center and a conserved methionine residue at the carboxy-terminus that forms the characteristic “methionine spin” (14).

The putative adhesion functions of adamalysins arise from the disintegrin domain and *cys-rich* domain. In addition, ADAMTSs also contain a variable number of TSP-1 motifs that are involved in interactions with multiple factors of the ECM. Functionally, adamalysins are involved in fundamental processes such as cellular interactions or intracellular signaling (15, 16).

**Figure 2:**
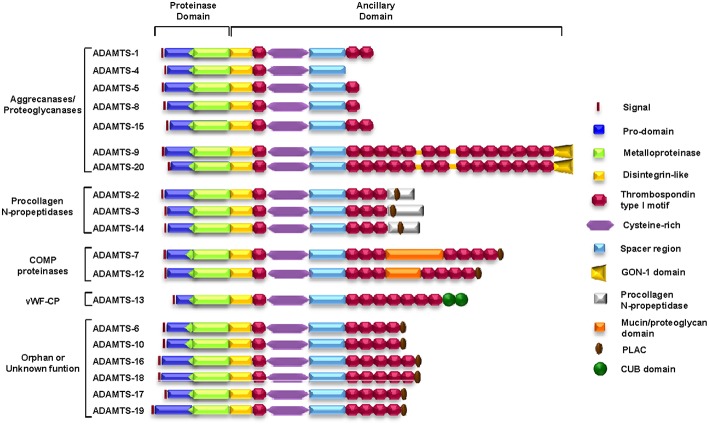
Schematic representation of the structure and motif composition of the ADAMTS family of ECM proteins. COMP, cartilage oligomeric matrix protein; vWFCP, von Willebrand Factor cleaving protease.

A total of 19 ADAMTSs have been identified in mammals (17) (Figure 2). The structural organization of ADAMTSs consists of a metalloprotease domain characterized by an aspartic residue at the end of the consensus Zn^2+^-chelating motif HExxHxxGxxHD, a disintegrin domain, a spacer domain, a mucin-like domain and several TSP-1 domains. One TSP-1 domain is located between the disintegrin domain and the *cys-rich* domain, and the rest reside in variable numbers at the carboxy-terminus of the protein. Other motifs, such as a GON-1 motif, are found in some members of the family, such as ADAMTS-9 and ADAMTS-20, and a cubillin (cub) motif is found only in ADAMTS-13 (18–20). The carboxy-terminal region of ADAMTSs is extraordinarily variable and gives these enzymes many of their unique properties. All domains in this region are mainly involved in adhesion and anchorage functions and profoundly affect the manner in which the protein interacts with its substrates as well as with other components of the ECM or the cellular surface. Notably, the term *orphan* ADAMTS, employed by Porter et al. (17) in 2005 to refer to ADAMTSs with no known substrate or function, can still be used today to refer to some members of the family.

## Fibulin-1 and its associations with ADAMTSs

The gene coding for fibulin-1, FBLN-1, also known as BM-90, was identified in 1989 as the first member of the fibulin family (21). FBLN-1 gene, located at human chromosome position 22p13, contains 20 exons and undergoes splicing to produce four mRNA variants. Thus, four different forms of fibulin-1 can be identified: fibulin-1A, fibulin-1B, fibulin-1C, and fibulin-1D. They differ in both the length and amino acid sequence of their terminal fibulin-like domain (22, 23).

In relation to tumor processes, fibulin-1 shows a dual function, since both oncogenic and tumor-suppressive properties have been described for this protein. For example, low expression of fibulin-1 is associated with poor prognosis in gastric, colon, and lung cancers (24–26). In other studies, epigenetic downregulation by hypermethylation has been described in various cancers, such as gastric, renal, colorectal, hepatocellular, and bladder cancers (27–31). However, fibulin-1 expression in the stroma is associated with increased malignancy in ovarian and breast cancers (32). Thus, the ambiguous function associated with fibulin-1 in tumor-related processes has several possible explanations: posttranslational modifications, such as the proteolytic processes affecting fibulin-1 in mammary tumors; alternative splicing, which generates the different forms of fibulin-1; interaction of fibulin-1 with different components of the ECM; or even the areas where fibulin-1 is expressed [stromal vs. tumor cells; (22, 33–35)].

ADAMTS-1 is an ECM component that can interact with fibulin-1 (36). ADAMTS-1 was the first identified member of the ADAMTS family, and its activation is mediated by a furin-like enzyme, leading to the elimination of the pro domain. Once activated, the enzyme may undergo a secondary processing event that separates the catalytic subunit from the thrombospondin repeats (37).

Like fibulin-1, ADAMTS-1 has also been implicated to participate in tumor processes with a dual function. Initially, antiangiogenic properties were associated with ADAMTS-1 through the inhibition of vascularization induced by basic fibroblast growth factor (bFGF) in corneal pocket assays. In addition, ADAMTS-1 blocked the angiogenesis process promoted by vascular endothelial growth factor (VEGF) in chorioallantoic membrane assays (38, 39). In addition, ADAMTS-1 can block tumor growth *in vivo* by sequestering VEGF165 through its carboxy-terminal region, which contains the final two TSP-1 motifs (40). In stark contrast, different studies have also demonstrated that this enzyme may elicit tumor-protective properties. For instance, ADAMTS-1 can promote tumor growth and progression in both breast and ovarian cancers (41–43) and can induce pathological angiogenesis through versican degradation (44). Additionally, this metalloprotease has been described to participate in a stromal reaction directed toward recruiting fibroblasts to participate in tumor growth (45).

**Figure 3:**
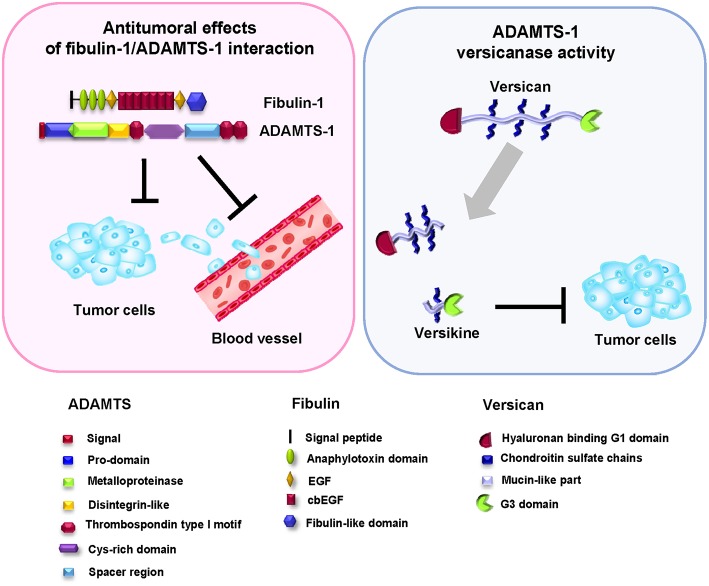
Antitumor effect of the fibulin-1/ADAMTS-1 interaction. Left, the fibulin-1/ADAMTS-1 interaction and its effects on tumor development. Right: ADAMTS-1 shows versicanase activity, which is potentiated by the presence of fibulin-1. Versikine, a byproduct of versican degradation, also shows antitumor properties. Bottom, protein motifs of ADAMTS, fibulin, and versican.

These apparently contradictory results can be explained in different ways. The pro- or antitumor effects have been suggested to be conditioned by the different fragments derived from autolysis of the enzyme (38). Other factors that may also affect the balance between the pro- or antitumor properties of ADAMTSs are the interaction with other components of the ECM or even the formation of bioactive cleavage products (46). Fibulin-1 binds to ADAMTS-1 through the last three replicates of the fibulin-like domain, similar to the TSP-1 motifs found in the carboxy-terminus of ADAMTS-1 (Figure 3); this interaction was confirmed by various *in vitro* and *in vivo* biochemical assays (36). The presence of fibulin-1 increases the catalytic activity of ADAMTS-1 to degrade aggrecan, resulting in the generation of two specific proteolytic fragments of 250 and 65 KDa. In fact, the level of fibulin-1 and an increase in aggrecan degradation by ADAMTS-1 are positively correlated. Fibulin-1 acts through altering the conformation of the aggrecan molecule and thus facilitating its proteolytic processing by the ADAMTS-1 metalloprotease (36). Versikine is a recently described bioactive product derived from the specific proteolytic degradation of versican (Figure 3) (47). Versikine participates in cellular functions in cancer development by regulating processes such as the apoptosis and migration of immune cells (48–50).

Versican proteolysis favored by the action of fibulin-1 as a cofactor of ADAMTS family members also occurs in interdigital web regression in mammals. Versican, a widely distributed proteoglycan in embryonic ECM, has been shown to interact with fibulin-1 through its carboxy-terminal G3 domain (51). Similar to its relationship with ADAMTS-1, versican is also a known substrate of ADAMTS-5 (52). Interdigital web regression requires the removal of not only interdigital cells, which occurs by apoptosis, but also the ECM (48). In this sense, and considering all these components of the ECM, ADAMTS-5 is the most active known versicanase among the proteases participating in the interdigital web regression process. In a similar way to that occurring in cardiogenesis or tumor processes, versican degradation by proteolysis is a critical event in web regression. Fibulin-1 regulates this process by enhancing the proteolytic activity of ADAMTS-5 and increasing the generation of a 70 kDa fragment from versican during interdigital tissue (IDT) regression. Finally, this fragment of versican is located in the population of cells destined to undergo apoptosis. This finding suggests that fibulin-1 acts as a cofactor for ADAMTS-5 in IDT regression (48).

In a recent study, the interaction between fibulin-1 and ADAMTS-1 was associated with antitumor effects in breast cancer cell lines (35). In fact, over-expression of exogenous ADAMTS-1 and fibulin-1 resulted in a reduction in the invasive and migratory phenotypes of these cell lines. In addition, the presence of both proteins also reduced the mammosphere formation capacity in *in vitro* experiments. In contrast, in the absence of fibulin-1, ADAMTS-1 shows a protumor function, indicating that the fibulin-1/ADAMTS-1 interaction could be considered a good prognostic factor in mammary tumors.

Fibulin-1 and ADAMTS-1 also seem to play crucial roles in other physiological events, such as the corneal formation or cardiogenesis (53, 54). By microarray analysis, fibulin-1 and ADAMTS-1 expression was detected in primary and immortalized human corneal fibroblasts (CHN), along with other components of the ECM known to interact with both proteins, such as nidogen-1 and aggrecan. The participation of fibulin-1 in cell migration was demonstrated after siRNA transfection of CHN cells, but cell adhesion was not affected (53). However, fibulin-1 participates in cardiogenesis by its high expression at sites of epithelial-mesenchymal transformation (EMT), in the endocardial cushion, in the coronary vessels and in the embryonic myocardium (55). Embryonic expression of fibulin-1 is considered transient in most tissues and disappears during development. However, fibulin-1 regulates versican-dependent events during ventricular morphogenesis (56). Versican is a proteoglycan essential for the formation of the endocardial cushion mesenchyme through EMT. As described for cancer development, ADAMTS-1 is an important factor in regulating versican activity through versican proteolysis events. Here, versican proteolysis results in the release of a 70 kDa fragment of versican, which is fundamental in the development and differentiation of the endocardial cushion mesenchyme (54). ADAMTS-1 as well as its cofactor fibulin-1, is expressed during early heart development (55, 57). The expression of both proteins suggests that fibulin-1 regulates the whole process of morphogenesis by enhancing the proteolytic activity of ADAMTS-1 toward versican and thus promoting the proliferation of cardiac cushion cells (55, 58, 59).

## Associations of Fibulin-2 with ADAMTSs

Fibulin-2 was discovered in 1993 from sequence analysis of cDNA clones obtained from a mouse fibroblast library. The FBLN2 gene is located at the p24-p25 region of human chromosome 3 and in the D-E band of mouse chromosome 6. Fibulin-2 possesses two distinct domains called the *cys-rich* and *cys-free* segments within its N-terminal region (60). This wide variety of domains serves to establish interactions with multiple ECM and cell surface components. For example, fibulin-2 can interact with aggrecan, nidogen, fibronectin, and perlecan, etc., as well as with cellular integrins (61–64), thereby contributing to the maintenance of extracellular structures such as basement membranes and elastic fibers (10).

The complexity of the functions of fibulin-2 is also evident in pathological conditions such as cancer (11, 65). As with fibulin-1, opposing functions have been described for fibulin-2. For example, protumor effects are associated with fibulin-2 in pancreatic cancer as a consequence of its interaction with type I transmembrane glycoprotein MUC4. This interaction might disrupt the integrity of the basement membrane, and thus promote the metastatic process (66). However, the antitumor properties of fibulin-2 are the most well-known, for example, in nasopharyngeal carcinoma, where fibulin-2 acts as an antiangiogenic factor (67), or in breast cancer, where the reduction in fibulin-2 expression facilitates tumor progression, increasing cellular migratory and invasive properties (68). In addition, the human gene encoding fibulin-2 is epigenetically silenced in acute B-cell lymphoblastic leukemia (69). Collectively, these studies show the importance of fibulin-2 in tumor development. However, the reason for this dual role in cancer is again believed to be due, similar to fibulin-1, to other factors such as posttranslational modifications, interactions with other components of the ECM, fibulin-2 site of expression within the cancerous tissue or even the clinical grade or stage of the tumor (70, 71).

**Figure 4:**
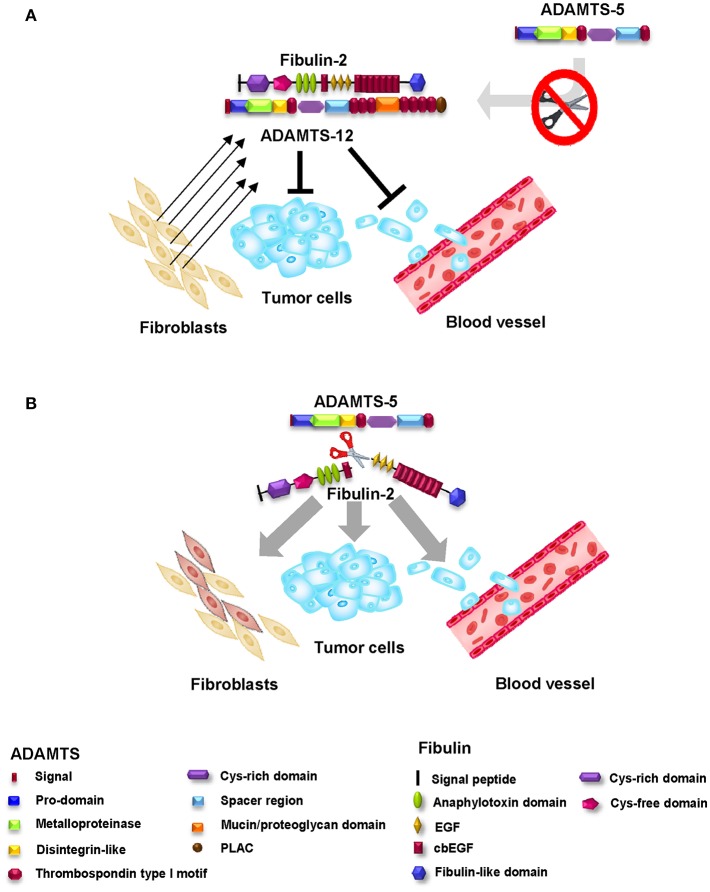
Antitumor effect of the fibulin-2/ADAMTS-12 interaction. **(A)** The fibulin-2/ADAMTS-12 interaction enhances the antitumor effect of both proteins in breast cancer by reduction of the invasive and migration capacities and the mammosphere formation capacity of breast cancer cells. In addition, this interaction protects fibulin-2 against degradation by aggrecanases (mainly ADAMTS-5). **(B)** Aggrecanases (ADAMTS-4 and ADAMTS-5) are able to proteolitically process fibulin-2 and thus, abolish fibulin-2 antitumoral function. The fibulin-2 proteolysis results in an increase of the invasive and migration capacities and mammosphere formation capacity of breast cancer cells. Bottom, protein motifs of ADAMTSs and fibulin-2.

Previous studies from our laboratory revealed that fibulin-2 is a molecular partner of ADAMTS-12 (72) (Figure 4A). ADAMTS-12 is a secreted metalloprotease associated with important roles in tissue remodeling, inflammation, cell migration, and adhesion (73–75). Regarding tumor-related processes, ADAMTS-12 has also been implicated to have a dual effect, described as a protumor factor as well as an antitumor factor. The participation of ADAMTS-12 with an antitumoral function has been described in colorectal carcinomas with silencing of the ADAMTS12 gene promoter by hypermethylation (76). This function is also suggested by the inhibition of the scattering of Madin-Darby canine kidney (MDCK) cells in the presence of HGF (75). MDCK cells in the presence of HGF experience both, a scattering effect and a tubulogenesis process, that ends in the formation of invasive extensions characteristic of a partial epithelial-mesenchymal transition (EMT) (77). Thus, abolishing this event by the presence of ADAMTS-12 can be considered as an antitumoral effect of this metalloprotease. Finally, studies with ADAMTS-12-deficient mice also indicate this antitumor phenotype (78). By using a model of induced tumors after malignant keratinocytes transplantation, the absence of ADAMTS-12 in *Adamts12*^−/−^ mice results in an increase in the angiogenic response and in tumor growth when compared with wild type mice. Furthermore, capillary outgrowth from mouse aortic rings cultured on a collagen gel was greater in aortic explants from *Adamts12*^−/−^ mice. The addition of conditioned culture medium obtained from cells that express ADAMTS-12 is able to suppress this capillary outgrowth also suggesting an antitumoral role for this protease (78).

The interaction between fibulin-2 and ADAMTS-12 occurs between the spacer-1 region of ADAMTS-12 and the carboxy-terminus of fibulin-2 (72). This interaction enhances the antitumor effect of both proteins in breast cancer, reducing the invasive capacity, migration, and the mammosphere formation capacity of the cells. In this context, where both proteins are exogenously over-expressed, the fibulin-2/ADAMTS-12 complex not only affects the cellular properties of breast cancer cell lines but also diminishes the growth of subcutaneous tumors in mice (72).

Another obvious demonstration of the importance of fibulin-2 within the tumor microenvironment is derived from the observation that it can be cleaved by the metalloproteases ADAMTS-4 and, especially, ADAMTS-5 (79) (Figure 4B). ADAMTS-4 and ADAMTS-5 are enzymes closely related to bone pathologies such as osteoarthritis due to their high aggrecanase activity (80, 81). Regarding tumor-related processes, both ADAMTS-4 and ADAMTS-5 are associated with a protumor role. In fact, the presence of both enzymes increases the invasive potential of glioblastoma cancer cells through brevican degradation (82, 83), and high levels of both ADAMTSs are related to an increase in the tumorigenic potential of ovarian carcinomas (43). Previous works have revealed that fibulin-2 can be proteolytically processed by MMPs and serine proteases involved in tissue remodeling (84). Our data regarding the cleavage of fibulin-2 by ADAMTS-5 strengthen the tumor-protective role of fibulin-2 in breast cancer. In fact, cleaved fibulin-2 is associated with an increase in the invasive phenotype of breast cancer cells; in addition, this processing can modify the nature of the surrounding fibroblasts by conferring protumor properties (79). However, there is a balance between the pro- and antitumor effects of fibulin-2 that seems to depend mainly on ADAMTS-12, which—as previously mentioned—interacts with fibulin-2, affording protection against cancer. However, in addition, this protective effect is extended by protecting fibulin-2 from degradation by ADAMTS-4 and ADAMTS-5. These effects highlight the importance of both fibulins and ADAMTSs as modifiers of the tumor microenvironment.

## Fibulin-3 and ADAMTS-5

Fibulin-3, also known as EFEMP1, is a protein located in the ECM of elastic tissues (12). Fibulin-3 may act as a regulator of cell growth and could influence the development of tumors. In support of these roles, fibulin-3 expression is increased in transformed cell lines compared to that in normal controls (85). In addition, when fibulin-3 mRNA is microinjected into normal human diploid fibroblasts (HDF normal cells), they undergo an increase in the rate of DNA synthesis compared to that in cells that have not been microinjected (65). However, fibulin-3 is absent from normal brain tissue (12, 86) and is downregulated in several types of solid tumors (87, 88). Surprisingly, fibulin-3 is upregulated in gliomas, where it promotes tumor growth and invasion (88). Recent results suggest that fibulin-3 is also upregulated in some highly metastatic tumors, where it correlates with the progression of these tumors toward the invasive phenotype (89, 90). Fibulin-3 participation in cancer implies different mechanisms. For example, fibulin-3 overexpression can be associated with a malignant phenotype and poor prognosis of cervical carcinoma. In this case, fibulin-3 promotes EMT by activation of the PI3K-Akt-mTOR signaling pathway (91). In osteosarcoma, fibulin-3 upregulation was positively correlated with poor prognosis and is able to promote cell invasion and metastasis by inducing EMT and activating the Wnt/β-catenin signaling pathway (92). Furthermore, fibulin-3 also stimulates migration and invasion of HCT116 cells through mechanisms involving p38α MAPK activation (93). In other cases, high levels of fibulin-3 are responsible for inhibiting EMT as well as migration, invasion and endothelial permeability processes in breast cancer microenvironment through the TGF-β mediated pathway (94). In lung cancer, fibulin-3 downregulation is required to promote invasion and metastasis by Wnt/β-catenin activation and MMP-7 expression (95). In summary, fibulin-3 participation in cancer might depend on the involved pathways, protein-protein interactions and tumor microenvironment.

A proposed mechanism by which fibulin-3 acts in tumor-related processes is as a signal to promote cell invasion and tumor dispersion. Consistent with this proinvasive role, CNS-1 glioma cell-induced tumors that overexpress fibulin-3 have significantly upregulated levels of mRNAs coding for several matricellular proteases, including MMP-2, MMP-9, and ADAMTS-5, all of which are involved in pericellular ECM degradation, glioma invasion, and intracranial tumor dispersion. In addition, cultured CNS-1 cells overexpressing fibulin-3 exhibited significant positive regulation of the same metalloproteases at the mRNA and protein levels as well as a strong increase in metalloprotease activity, suggesting a direct regulatory effect of fibulin-3 on the expression of metalloproteases such as ADAMTS-5 and the proteolytic activity observed in glioma cells (88).

## Conclusion

The ECM that forms any given tissue is a determinant for numerous cellular processes. Certain components can modify this environment, leading to the generation of organs or to the development of pathologies, such as cancer. Thus, the ECM plays an important role in cellular behavior and, as a consequence, can be a force in remodeling the cellular environment and, consequently, cellular properties.

**Figure 5:**
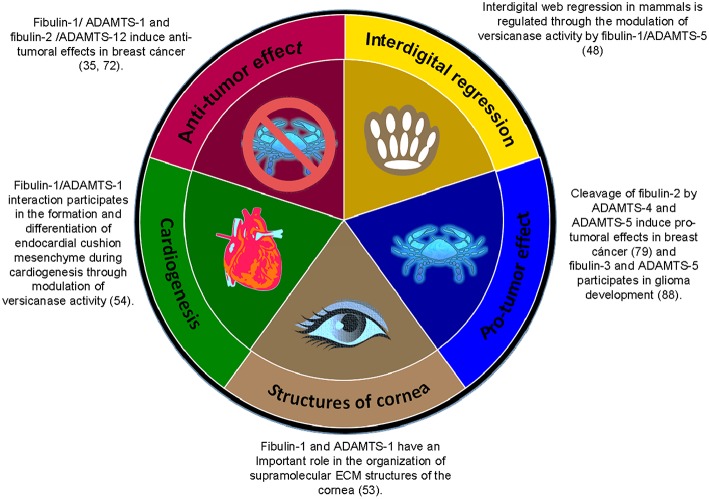
Summary of the associations between members of the fibulin family and members of the ADAMTS family of ECM proteins in the regulation of various physiological and pathological processes.

In this review, we focused on the relationship between two families of ECM proteins: fibulins and ADAMTSs (Figure 5). Among the fibulins, fibulin-1 is the best characterized member, with the greatest number of described partners. In fact, this protein seems to play a more relevant role than its related family member fibulin-2, considering the characteristics of fibulin-1-deficient mice. Fibulin-1 knockout mice do not survive beyond birth because of bleeding in the muscle, skin and perineural tissues (96) while fibulin-2 deficient mice are viable, fertile and do not exhibit apparent phenotypic defects (97). The function of fibulin-2 seems to be compensated for by an increase in fibulin-1 expression, which, in turn, compensates for the absence of fibulin-2. Fibulin-1 and fibulin-2 can interact with two related members of the ADAMTS family of proteases, ADAMTS-1 and ADAMTS-12, respectively. These interactions can modify the protumor properties exhibited by ADAMTS-1 and ADAMTS-12 in breast cancer cells. Consequently, the simultaneous presence of fibulin-1 and ADAMTS-1 or of fibulin-2 and ADAMTS-12 could be considered a good prognostic factor in breast cancer. Moreover, fibulin-1 is a known cofactor of the proteolytic activity of ADAMTS-1 toward some of its substrates, such as versican. Fibulin-1 is also implicated with ADAMTS-5 in other physiological processes such as interdigital web regression by modulation of its versicanase activity. However, ADAMTS-5 also cooperates with fibulin-3 to promote cell invasion in gliomas through proteolytic degradation of ECM components. Finally, ADAMTS-5 and ADAMTS-4 interact with and can degrade fibulin-2. Interestingly, the degradation of fibulin-2 by either ADAMTS-4 or ADAMTS-5 is blocked by the presence of ADAMTS-12 and seems to be a target for the protective role of the fibulin-2/ADAMTS-12 interaction in breast cancer (Figure 5). Collectively, these studies demonstrate the importance and increasing number of interactions between members of both of these families of secreted multidomain proteins. The exploration of new associations between fibulins and ADAMTS would help to shed light on not only the maintenance of tissue homeostasis but also the involvement of these proteins in pathological processes.

## Author Contributions

All authors listed have made a substantial, direct and intellectual contribution to the work, and approved it for publication.

### Conflict of Interest Statement

The authors declare that the research was conducted in the absence of any commercial or financial relationships that could be construed as a potential conflict of interest.
